# Novel biotechnological approaches for monitoring and immunization against resistant to antibiotics *Escherichia coli* and other pathogenic bacteria

**DOI:** 10.1186/s12917-020-02633-8

**Published:** 2020-11-02

**Authors:** José E. Belizário, Marcelo P. Sircili

**Affiliations:** 1 Department of Pharmacology, Institute of Biomedical Sciences, University of São Paulo, Av. Lineu Prestes, 1524, São Paulo, SP CEP 05508-900 Brazil; 2 Laboratory of Genetics, Butantan Institute, Av. Vital Brazil, 1500, São Paulo, SP CEP 05503-900 Brazil

**Keywords:** Bacterial vaccines, Microbiomes, Probiotics, Antibiotics, VOCs, And FAMEs

## Abstract

The application of next-generation molecular, biochemical and immunological methods for developing new vaccines, antimicrobial compounds, probiotics and prebiotics for zoonotic infection control has been fundamental to the understanding and preservation of the symbiotic relationship between animals and humans. With increasing rates of antibiotic use, resistant bacterial infections have become more difficult to diagnose, treat, and eradicate, thereby elevating the importance of surveillance and prevention programs. Effective surveillance relies on the availability of rapid, cost-effective methods to monitor pathogenic bacterial isolates. In this opinion article, we summarize the results of some research program initiatives for the improvement of live vaccines against avian enterotoxigenic *Escherichia coli* using virulence factor gene deletion and engineered vaccine vectors based on probiotics. We also describe methods for the detection of pathogenic bacterial strains in eco-environmental headspace and aerosols, as well as samples of animal and human breath, based on the composition of volatile organic compounds and fatty acid methyl esters. We explain how the introduction of these low-cost biotechnologies and protocols will provide the opportunity to enhance co-operation between networks of resistance surveillance programs and integrated routine workflows of veterinary and clinical public health microbiology laboratories.

## Background

**Fig. 1:**
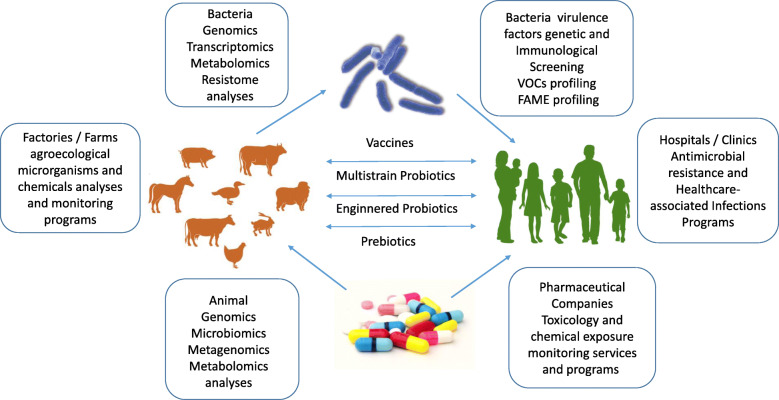
Innovative biotechnological approaches and programs to reduce the veterinary use of antibiotics, the ability of bacteria to resist antibiotics, and bacterial spread into animals and humans, within the responsibilities of research laboratories, farms, factories, pharmaceutical companies, hospital and clinics. Adapted from [9]. Images on figure are of public domain and non-copyrighted

In many South American countries, more than two-thirds of all antimicrobials produced are administrated to animals, not human patients, mainly for growth promotion, treating animal disease and prophylaxis [1, 2]. Indiscriminate use of antimicrobials may select for drug-resistant pathogens as well as for mobile genetic elements (plasmids, bacteriophages) carrying antibiotic resistance genes (ARGs) to both human and nonhuman animal pathogens. In addition, antibiotic overuse contributes to the spread of antimicrobial-resistant organisms and increases the risk factor of infections and diseases in both animals and humans [3]. Bacteria commonly found in the poultry industry include *Campylobacter spp*., *Staphylococcus spp*., *Salmonella spp*., *Clostridium perfringens* type A, *Enterococcus faecalis*, and *Escherichia coli* [4, 5]. Dietary supplementation with broad-spectrum antimicrobials such as bacitracin prevents the risk of necrotic enteritis caused by *C. perfringens* in broiler chickens. However, this provides the opportunity for increased antibiotic resistance. Transmission of antimicrobial-resistant bacteria can occur through direct contact, contaminated water, air, environment, and food [6–8]. The increase in the extended-spectrum cephalosporin resistance in *Salmonella* isolated from retail chicken is the most important public health problem in developing countries [6–8]. Furthermore, workers exposed to antibiotics and potential pathogens may disseminate antimicrobial-resistant bacteria in their community, contributing to evolutionary trajectories of evolving microbes and failures in antibiotic treatment outcomes [6–8]. Figure 1 illustrates a framework of biotechnological, antimicrobial stewardship and public health programs with the aim of reaching decisions regarding the adoption of these basic and applied technologies in study design and economic evaluations of the global challenges in the rational use of antibiotics in human beings and animals.

Animal fecal microflora harbor a vast reservoir of antibiotic-resistance genes, which are predominantly acquired by human commensal and pathogenic bacteria [10, 11]. The abuse and unreasonable utilization of antimicrobials for clinical, agricultural and pharmaceutical purposes can lead to a selective pressure in metabolic and oxidative biochemical processes for DNA mutation [1, 12]. Sublethal bactericide pressure can increase mutagenesis in hypermutator bacterial strains and the acquisition of resistance genes [12]. Several antibiotics have been used in feed or water at sub-therapeutic levels for growth promotion for over 50 years [13, 14]. Sub-therapeutic in-feed antibiotics such as erythromycin, penicillin, gentamicin, neomycin, tetracyclines, bacitracin methylene disalicylate and virginiamycin (streptogramin A and B) are commonly used for growth promotion or prophylaxis in poultry production [15]. Moreover, sublethal doses of bactericide antibiotics promote the specialization of various different bacteria genotypes within the whole heterogeneous bacterial population. For example, sublethal doses of norfloxacin over 10 days greatly increase resistant isolates from the *E. coli* population, which release indole, a signaling molecule that protects less resistant isolates. The rise of extended-spectrum beta-lactamase (ESBL)-producing *E. coli* in food-producing animals poses significant challenges to clinicians and negatively impacts patient healthcare in hospitals [8, 16]. Studies of fecal resistome indicated that diverse genera of *Enterobacteriaceae,* including *E. coli*, *Klebsiella*, *Shigella*, and S*almonella* have acquired different antibiotic resistance genes [17, 18]. Remarkably, *E. coli* strains are the predominant (83%) bacterial host of genes for resistance to tetracycline, aminoglycoside, macrolide, lincosamide, streptogramin, β-lactam and sulfonamides [8, 16].

Airborne cross-transmission of pathogenic bacteria between animals and workers in swine and dairy facilities has been well characterized through surveillance programs [19]. These studies have reinforced the necessity for implementation of surveillance programs to detect the spread of zoonotic diseases between animals and the population [19]. Poultry workers in general acquire *Campylobacter* through orally transmitted water droplets [19, 20]. However, the presence of antibiotic-resistant bacteria in biological aerosols is largely not understood. Therefore, innovative approaches are necessary to monitor and collect specific solid and organic volatile compounds that are produced and released by large animal feeding and factory facilities.

Poultry facilities are associated with a high production of dust, gases, and odors [4, 20]. In poultry bioaerosols, bacteria exist suspended freely in the air as well as attached to dust particles [4, 20]. The presence of antimicrobial genes in poultry bioaerosols suggests that poultry farmers may be potential nasal carriers of antimicrobial-resistant bacteria, which could potentially be transmitted to susceptible persons [14]. Swine workers are also nasal carriers of resistant bacterial species [14] Pathogenic bacteria carrying tetracycline-resistance genes were detected in bioaerosols from swine confinement buildings and nasal swabs of swine workers [21]. Finally, respiratory pathogens such as *Haemophilus influenzae*, *Streptococcus pneumoniae*, and *Moraxella catarrhalis* are commonly detected in blood, cerebrospinal fluid, higher and lower respiratory tract, urine, and wound or pus of farm animals and humans. Substantial investments are needed to develop the innovative vaccines for these new targets at the commercial scale. In this opinion article, we will update specifically on potential vaccines in development against avian pathogenic *E. coli* strains and novel engineered vaccine probiotics based on *Lactobacillus spp.* and yeasts. In the second part, we will describe the technical and biotechnological discoveries that are being implemented for early identification of presence/absence of pathogenic bacterial strains, including those with acquired antimicrobial resistance based on the composition of volatile organic compounds (VOCs) and volatile fatty acids (FAMEs).

### Antibiotic growth-promoting effects

In-feed antibiotic supplementation exerts growth-promoting effects in farm animals, through a yet unclear mechanism [22]. Early studies demonstrated that the use of antibiotics promotes the suppression of the number and diversity of the normal gut bacterial flora and the production of certain microbial metabolites while increasing the concentration of critical nutrients [23] The large-scale systematic analysis of farm animal microbiomes has recently emerged. There are few studies addressing the long-term dynamics of the intestinal microbiota in relation to changes in energy absorption and/or food intake under antibiotic-induced growth treatment. Chickens fed with virginiamycin- and bacitracin-supplemented diets had reduced microbial diversity, with an increase in *Enterococcus* and *Lactobacillus spp.* and a decrease of *L. salivarius* [24]. The development of new generation genomic and metabolomic approaches has allowed analyses of the differences in intestinal microbiota composition and the amino acids, vitamins and metabolites they produce under diverse diets and antibiotic intervention [25]. Based on a metabolomics approach, Gadde and colleagues in a recent study proposed a mechanism by which dietary antibiotics may exert enhanced growth promotion in broiler chickens [24]. They showed that antibiotic treatment alters the host intestinal anti-inflammatory response in parallel with changes in the structure and diversity of the gut microbial community [24]. Antibiotic supplementation with bacitracin increased the production of polyunsaturated fatty acid (PUFA) levels in the chicken intestine. Fatty acids are incorporated into the membranes of leukocytes and serve as substrates for production of bioactive lipid mediators. Three biosynthetic routes working independently or in concert produce prostaglandins, leukotrienes, lipoxins and resolvins, which are well known mediators of pro- and anti-inflammatory effects during the early and late phases of an inflammation. Dietary supplementation with omega-3 PUFAs such as eicosapentaenoic acid (EPA) and docosahexaenoic acid (DHA) are known to reduce inflammatory reactions [26]. Considering that the antibiotics are given in absence of inflammation, studies have been carried out to identify which are the cells and mediators responsible for antibiotic effects. Previous studies have identified that neutrophils are targeted and killed by apoptosis after macrolide tilmicosin treatment [27]. Along inflammatory response and diverse disease setting, lipoxins act as potent anti-inflammatory mediators and promote neutrophil killing in the resolution phase of an inflammation [26]. Do these local lipid mediators serve as effectors of antibiotics? In this way, it is quite possible that resolvins and protectins derived from DHA could also promote the clearance of neutrophils from mucosal surfaces [26]. This remains to be further investigated.

### Synthetic microbiomes and engineered vaccine probiotics

Dysbiosis of healthy gut microbiota plays a critical role in the dysregulation of microbial ecology that favors colonization of pathogenic bacterial strains and diseases [28]. The literature is replete with studies that characterize the structure and diversity of the gut microbiome, through sequencing the 16S RNA gene, and the correlation with different nutritional states, including obesity and malnutrition, as well as many metabolic and inflammatory disorders [29]. The current hypothesis is that the size of or shift in the Prevotella enterotype/Bacteroides enterotype ratio determines the variations in the production of short chain fatty acids (SCFAs) acetate, propionate, butyrate and lactate [29–32]. These molecules are critical metabolic substrates for gluconeogenesis and lipogenesis that drive obesity [32]. SCFAs, particularly, propionic and butyric acids, may directly prevent the low-grade inflammatory response observed in some obese populations [32, 33]. Bacteriotherapy or fecal microbiota transplantation have been in clinical practice over centuries, in particular in the treatment of refractory cases of *Clostridium difficile* colorectal infection after antibiotic therapy [28]. In this context, competitive exclusion (CE) products derived from healthy specific-pathogen-free chickens and administered by crop gavage have been used as feed supplements in broiler chickens [34–36]. This type of bacteriotherapy effectively increases the resistance and protects chickens from bacterial infections, notably salmonellosis [34]. Freeze-dried CE preparations are manufactured and commercialized in many countries [37]. Simple fecal transplantation or microbiota transfer can reproduce obesity or leanness in animal models [29, 30]. Nonetheless, since host-associated microbes perform several important functions, in particular metabolic and immunological functions, the question is: How many strains or species belonging to Firmicutes, Bacteroidetes and Actinobacteria phyla are needed for a minimal microbiome that could exert all microbial functions? Synthetic microbial communities with specific desired metagenomes and function can be designed and manufactured reproducibly [38]. For instance, bacterial species or consortia that are associated with resistance to infection could be designed. Therefore, there is great expectation of the development and application of future engineered synthetic microbiomes in which a personalized (or animalized) diet will improve healthy animal growth.

The World Health Organization (WHO) has defined probiotics as live organisms that, when administered in adequate amounts, confer a health benefit to the host. Probiotics are, in general, members of the microbiota. Extensive basic and clinical studies with lactic acid bacteria strains such as *Bifidobacteria spp., Bacteroides* and *Akkermansia spp.* have provided strong evidence of their health benefits. This is achieved through multiple mechanisms and effector molecules [32, 39, 40]. In-feed supplementation with *Bacillus*, *Bifidobacterium*, *Enterococcus*, *Lactobacillus*, *Streptococcus*, *Lactococcus spp*. and *Yeast Saccharomyces* have been given as probiotics to inhibit infection of enteric pathogens and mitigate antibiotic-associated diarrhea [41–43]. Recently, probiotics based on *L. lactis* and *Lactobacillus spp.* have been explored as delivery vectors of therapeutics and antigens expressed by virus and bacteria [44]. Novel engineered probiotics based on yeast strains, mainly *Saccharomyces boulardii*, have been constructed to secrete multi-specific and single-domain antibodies directly targeting bacterial virulence factors, in particular, enterotoxins [45]. Specifically, *S. boulardii* was engineered to express one or more antigens aiming to improve the immune response to *C. difficile* and *Campylobacter jejuni* infection [46]. Furthermore, these probiotic strains can be engineered, or reprogrammed, for heterologous gene expression of amino acids, peptides and antimicrobial compounds to combat multiple pathogens [38, 43, 44] Yet, fundamental questions remain: Does the make-up of the gut microbiome impact the development of body carcass from the perspective of quantitative bioenergetics? How might we monitor and manipulate the gut microbiome to optimize the complex diet–microbiome relationship and positively impact the host? These are some questions to be answered.

### Chicken *E. coli* vaccines

Enterotoxigenic *E. coli* (ETEC) infection is globally the most common cause of serious diarrheal illness [47]. Active vaccination (long lasting) and passive immunization with antibodies (short-lived) are the most appropriate immunological methods to prevent diseases caused by bacterial pathogens. Live vaccines consisting of attenuated strains of key serotypes is an alternative way to avoid the continued use of antibiotics in veterinary medicine [48]. In Brazil, the world’s largest exporter of chicken meat, *Enterobacteriaceae* species are responsible for more than 45% of condemned poultry carcasses [49]. *E. coli* is considered a major source of the spread of antimicrobial resistance to other bacteria, which are mainly mediated by exchange of genes, integrons, transposons, and plasmids [47, 48]. Avian *E. coli* strains are clearly differentiated with respect to phylogenetic relationships, common virulence factors and pathogenicity to animals and humans [50–52]. Single isolates within species from different geographical areas vary profoundly in terms of virulence factor traits and antibiotic-resistance genes. More knowledge of the molecular basis of this variability will help in the design of new rational approaches to monitoring pathogenicity and immunogenicity of new bacterial mutants.

Many ETEC strains colonize the gastrointestinal tract of birds and mammals where they live in a commensal relationship [47, 48]. The avian pathogenic *E. coli* (APEC) strains cause systemic septic colibacillosis, which is characterized by bloodstream invasion resulting in massive lesions in multiple internal organs and sudden death of birds. Extra-intestinal pathogenic *E. coli* (ExPEC) strains are commonly associated with nosocomial and community infections in humans and animals [49–53]. A larger number of ExPEC isolates from humans and animals of have been characterized based on their origins as follows: uropathogenic *E. coli* (UPEC), neonatal meningitis *E. coli* (NMEC) sepsis-associated *E. coli* (SEPEC), and avian pathogenic *E. coli* (APEC) [47, 48, 54]. It is interesting that ExPEC isolates from humans and chickens have similar virulence traits and common genes [47, 48, 54, 55]. A conserved set of 13 virulence-associated genes identified APEC isolates. The most studied are *fimC*, *astA*, *papC*, *tsh*, *fyuA*, *irp2*, *iucD*, *iss*, *hlyE*, *eaeA*, *vat*, *colV*, and *stx2f*. This set of genes has been used to confirm genotypically and phenotypically the relationships among pathogenic APEC isolates [56, 57]. The virulence genes for adhesins *fimH* (Type 1 fimbriae) and *tsh* (temperature-sensitive hemagglutinin), protectin *iss* (increased serum survival) and the iron acquisition system genes (iron-scavenging systems) are examples of genes that convert intestinal commensal into pathogenic *E. coli* (APEC). Many studies have supported the role of APECs as zoonotic and foodborne pathogens [50, 54]. Currently serotypes O1:K1, O2:K1 and O78:K80 are recognized as the most prevalent serotypes among more than 20 APEC subtypes identified in animal studies. However, despite the huge importance of APECs for bird health there are few studies searching for genes evolved in recombination and positive selection as well as specific virulence factors associated with traits and phenotypes [49–51]. More importantly, most the time these pathogenic strains are carrying plasmids that disseminate resistance to ampicillin, tetracycline, gentamicin, neomycin, sulfa trimethoprim, enrofloxacin and norfloxacin [5, 58]. ExPEC-commensal *E. coli* strains harboring multidrug resistance (MDR) have also been isolated in different poultry farms [52]. Therefore, continuous monitoring of bacterial resistance genes among *E. coli* ExPEC and APEC isolates from chicken meat products will aid the development of surveillance programs and new scientific strategies for food safety.

A series of studies was conducted by Dr. Wanderley Silveira’s group (UNICAMP, Campinas, Brazil) to identify molecularly the APEC strain SEPT362, serotype OR:H10, which was isolated from liver of a laying hen presenting clinical signs of septicemia [59–61]. Overall, the results suggested that the pathogenicity of APEC strain SEPT362 and its biological characteristics are determined by a set of genes controlling the type VI secretion system (T6SS). The authors generated three *E. coli* APEC mutant strains deficient in *hcp*, *clpV* and *icmF* genes of the T6SS and investigated their function in the SEPT361 *E. coli*-induced lethality in chicks [59–61]. Chicken infected with *E. coli* deficient in *hcp* and *clpV* genes had a survival rate of 93% on the first day, 68% on the third day, and 50% on the sixth day after infection. Vaccination with *E. coli* deficient in *clpV* gene only significantly increased survival rates of chicken over 30 days of experimentation. The pathogenicity was not significantly decreased with *E. coli* mutants of *icmF* gen*e*. On the other hand, it was observed that *icmF* gen*e E. coli* mutant reduced intracellular viability of *E. coli* in infected macrophages. Furthermore, all *E. coli* mutants showed a reduced biofilm formation capacity as compared to wild-type SEPT362 strain. Overall, the results suggest that *E. coli* SEPT362 deficient in *hcp*, *clpV* and *icmF* genes have enhanced safety, stability and the potential capacity as live or attenuated vaccine for future experimental study aimed at chicken vaccination against *E. coli*. Various attempts have been made to develop an effective vaccine against the respiratory form of APEC diseases in poultry [62, 63]. The enzyme 5-enolpyruvylshikimate-3-phosphate synthase (EPSPS), also designated as AroA, participates in the biosynthesis of aromatic amino acids. A live vaccine based on an *E. coli* mutant strain with *aroA* deleted has been used as a spray vaccine. This live-attenuated APEC O78 ΔaroA vaccine (Poulvac® *E. coli*) protected the manifestation of colibacillosis in chickens infected with homologous and heterologous APEC strains [64]. However, another study demonstrated that this vaccine was incapable, by itself, of inducing humoral immunity in turkeys. An efficient cellular immune response was only observed when the vaccine was administrated with strong co-adjuvants containing CpG nucleotides [65]. The quorum-sensing system (QS) plays important roles in the production of bacterial toxins, biofilm formation, and pathogenicity of APECs [66]. The *luxS* is a gene that codes for a S-ribosylhomocysteine lyase that catalyzes the substrates for production of the autoinducer 2 (AI-2), a furanosyl borate that coordinate AI-2 QS system involved in the density-dependent processes in Gram negative and positive bacteria. A live-attenuated APEC strain deficient in both *luxS* gene and *aroA* gene, which participates in the biosynthesis of aromatic amino acids, has been evaluated as potential vaccine candidate [67]. This double-mutant strain attenuated APEC virulence by more than six-fold as compared to the single-mutant strain DE17 [67]. Together, the results of these studies suggest that the virulence deletion approach is a worthy strategy to produce safe live and attenuated vaccines to prevent avian colibacillosis. More importantly, these studies predict that vaccination is an appropriate method for reducing the rise and spread of resistant *E. coli* APEC strains within animals and the community.

### VOCs

DNA/RNA/protein high-throughput sequencing and/or structural determination methods are powerful tools to give a deeper qualitative and quantitative insight into bacterial species and their resistomes [10–12]. Simple and cost-efficient methods for collecting exhaled breath and extracting DNA, proteins, peptides, metabolites and VOCs are in development for bacterial species and genera identification. Bacteria have distinct metabolic pathways compared to human cells; thus, their presence can be characterized by odor and/or release of inert VOCs [68]. Over 1000 VOCs emitted by bacterial and fungal strains categorized by classes and chemical structures have been cataloged [69]. The gastrointestinal microbiota produces a wide range of sulfur-containing gaseous substances, including hydrogen sulfide (H_2_S), which serves as a substrate for methane synthesis, methyl mercaptan, dimethyl sulfide, dimethyl disulfide and trisulfide. All these compounds are toxic and induce inflammation when in high concentration. The end products of anaerobic bacteria fermentation of oligosaccharides include succinate and a mixture of volatile fatty acids, mainly acetate, propionate, cis-2-methylcrotonate, 2-methylbutyrate and 2-methylvalerate, SCFAs, as well as alcohols, hydrocarbons, aldehydes, ketones and sulfated organic compounds [68, 70–72]. Many of these organic compounds can be detected in expelled air, feces and urine and explored for confirmation of pathogenic infection [68, 70–72]. Indole has been used clinically as a predictive biomarker of *E. coli* biofilm. Butyric acid is a biomarker for some *Clostridium* species, acetaldehyde for some *Staphylococcus* species, and long chain alcohols are important biomarkers for the detection of *Salmonella*. VOCs can discriminate pathogen interactions commonly observed in human clinical infections. For example, high levels of 2-methylbutylacetate and methyl 2-methylbutyrate, two known antimicrobial VOCs, were found only in a co-culture of *E. cloacae* and *P. aeruginosa* [73]. A systematic review of the literature identified 161 VOCs emitted during sepsis in children [68]. The author identified VOC signatures that allowed prediction of the presence of the Gram-positive bacteria *Staphylococcus aureus*, *Streptococcus pneumonaie* and *Enterococcus fecallis*, and the Gram-negative bacteria *E. coli*, *Pseudomonas aeruginosa* and *Klebsiella pneumoniae* [68]. Palma and colleagues, using a machine-learning algorithm, identified a group of 18 VOCs that could predict with accuracy and precision 11 species of bacteria, protozoa and fungi [74]. The growing international community of breath VOC researchers is conducting standardized large-scale studies to catalog endogenously derived volatile compounds originating from pathogens in culture and released in exhaled human breath, skin, urine, feces, and flatulence in healthy and diseased people as well as from plants, foods, and soil microorganisms [68, 71]. The mVOC database (http://bioinformatics.charite.de/mvoc) is one user-friendly platform for VOC-based retrieval. Such databases will help establish a powerful resource for investigation of the modulatory effects of VOCs in bacterial growth control and resistance to antibiotics.

Animal feeding operations produce a larger number of gaseous chemical compounds [75]. More than 100 VOCs were identified in the indoor and outdoor air, stable and road dust and soil samples from pig and cattle farms [75]. Acetic acid, butanoic acid, p-cresol, propanoic acid, pentanoic acids, phenol and hexanal were found in the indoor air of pig farms, and acetic acid, butanoic acid, propanoic acids, p-cresol, phenol and methyl esters of carboxylic acids in the indoor air of cattle farms [75]. Various chemical substances were identified using gas chromatographic analysis of VOCs captured by evacuated canisters and sorbent tubes distributed at critical points of the installations. Besides gas chromatography methods, various automated devices for the detection of VOCs have been developed using a wider array of sensor heads (eNose) and detection systems [76–78]. These specific sensor types can offer simple solutions for specific criteria in terms of selective detection of target VOCs in field applications. The speciation and levels of VOCs released in animal facilities and production stations include compounds formed by biotic and abiotic stress. Thus, there is opportunity for development and application of a range of biotechnological and chemical techniques to access the volatility and reactivity of VOCs with respect to opportunistic pathogens such as *Micrococcus*, *Bacteroides*, *Chryseobacterium*, *Pseudomonas*, and *Acinetobacter* and parasites specifically found in domestic animals. The creation of databases of microorganism-specific VOC signatures using such methodologies has great potential for tracking the presence and spread of antibiotic-resistant species dispersed through bioaerosols.

### FAMEs

The fatty acid composition of bacterial species is genetically conserved [79]. When esterified by an alkali-catalyzed reaction with methanol, the fatty acids become volatile enough for analysis by gas chromatography [80]. The analysis of the volatile fatty acids (VFAs), in their free acid form, or as fatty acid methyl esters (FAMEs) can be used to predict the presence of aerobic and anaerobic bacteria [80–82]. Gas chromatography/mass spectrometry (GC/MS) analysis of fatty acids between 9 and 20 carbons in length (microbial fingerprinting) is a conventional method to characterize genera and species of bacteria, especially non-fermentative Gram-negative organisms [82, 83]. The system for naming and counting carbons is from the carboxyl end (omega end). For example, terminally branched saturated FAMEs indicate the presence of Gram-positive bacteria and monounsaturated or hydroxylated FAMEs Gram-negative bacteria. Normally, polyunsaturated FAMEs are rarely encountered in bacteria. Over 1500 bacterial species have been identified based on their unique fatty acid profiles on commercial identification systems such as the Sherlock Microbial Identification System [80, 84]. In a recent publication, Roth and colleagues developed a GC method to characterize dental bacterial plaque via FAME profiling [85]. They successfully identified saturated and monoenoic FAMEs in the biofilm cultivated in vitro from 10 donors [85]. A machine-learning bioinformatics approach called phylogenetic learning has been used for FAME-based bacterial species classification [86]. FAME database [http://www.fame-bank.net] is an open platform that displays a taxonomic framework classifier based on FAME composition and bacterial 16S rRNA gene sequences [86]. Thus, we expect that the expansion of VOCs and FAMEs chemical signatures will improve our understanding of the role of participating bacteria from different genera and species as well as strains of the same species. These methods can potentially impact the speed and accuracy by which bacterial antibiotic-resistant species are identified and correctly treated.

## Conclusions and outlook

Global environmental changes and ecosystem impairments have contributed to the dissemination of bacterial pathogens from animals to humans and vice-versa. Despite progress in some countries, effective and coordinated strategies, such as antimicrobial stewardship and One Health strategy, for the containment of misuse of antimicrobials and the spread of antibiotic-resistant microorganisms have not been globally implemented [87]. The European Union has banned the non-therapeutic feeding of a number of antibiotics of human importance to farm animals. However, Europeans consume antibiotic-treated vegetables and meat products from countries that allow the use of antibiotics. Therefore, a global solution to these problems depends on the development of novel innovative biotechnologies to enhance co-operation with and between networks of public bacterial resistance surveillance programs. We need to be prudent and maintain rational prescribing programs in pro-growth, prophylactic and therapeutic use of veterinary antibiotics to eliminate existing resistant bacterial strains from a population and new ones that may arise in the future [88, 89]. More attention should be done in providing best practices for the prescription of antibiotics to farmers and veterinary personnel with confirmed presence of both animal and nosocomial antimicrobial resistant pathogens. In this way, we need to improve science through robust investments in the research, development, and implementation of stewardship programs in all outpatients and inpatients healthcare settings.

The next generation of live bacterial strains and engineered probiotics based on lactic acid bacteria and yeast as vaccine delivery vectors offer many attractive advantages over traditional recombinant vaccines. Rapid diagnostic methods for bacterial infections and airborne disease outbreaks in farm animals and communities are constant challenges faced by hospitals and health institutions in developing countries. Antibiotic stewardship programs are a key component in preventing the spread of antibiotic resistance in all healthcare facilities and across the world. Non-invasive and fast diagnostic tools based on breath biopsy hold great promise in identifying animal and human pathogens. The implementation and use of chromatography and mass spectrometry methods and chemical sensors in the clinical laboratory for determination of VOCs and FAMEs have immense potential in the development of biomarkers for the early diagnosis of infectious diseases. Finally, the application of these innovative methods will provide quantitative insights into the contribution of microbiota to farm animal growth and enable future studies on the interacting roles of diet and gut microbiome on animal physiology and health.

## Data Availability

Data sharing is not applicable to this kind of article.
